# HIV/AIDS Competent Households: Interaction between a Health-Enabling Environment and Community-Based Treatment Adherence Support for People Living with HIV/AIDS in South Africa

**DOI:** 10.1371/journal.pone.0151379

**Published:** 2016-03-10

**Authors:** Caroline Masquillier, Edwin Wouters, Dimitri Mortelmans, Brian van Wyk, Harry Hausler, Wim Van Damme

**Affiliations:** 1 Research Centre for Longitudinal and Life Course Studies (CELLO), University of Antwerp, Antwerp, Belgium; 2 Centre for Health Systems Research and Development, University of the Free State, Bloemfontein, South Africa; 3 School of Public Health, University of the Western Cape, Bellville, South Africa; 4 TB/HIV Care Association, Cape Town, South Africa; 5 Department of Public Health, Institute of Tropical Medicine, Antwerp, Belgium; Public Health Agency of Barcelona, SPAIN

## Abstract

In the context of severe human resource shortages in HIV care, task-shifting and especially community-based support are increasingly being cited as potential means of providing durable care to chronic HIV patients. Socio-ecological theory clearly stipulates that–in all social interventions–the interrelatedness and interdependency between individuals and their immediate social contexts should be taken into account. People living with HIV/AIDS (PLWHA) seldom live in isolation, yet community-based interventions for supporting chronic HIV patients have largely ignored the social contexts in which they are implemented. Research is thus required to investigate such community-based support within its context. The aim of this study is to address this research gap by examining the way in which HIV/AIDS competence in the household hampers or facilitates community-based treatment adherence support. The data was analyzed carefully in accordance with the Grounded Theory procedures, using Nvivo 10. More specifically, we analyzed field notes from participatory observations conducted during 48 community-based treatment adherence support sessions in townships on the outskirts of Cape Town, transcripts of 32 audio-recorded in-depth interviews with PLWHA and transcripts of 4 focus group discussions with 36 community health workers (CHWs). Despite the fact that the CHWs try to present themselves as not being openly associated with HIV/AIDS services, results show that the presence of a CHW is often seen as a marker of the disease. Depending on the HIV/AIDS competence in the household, this association can challenge the patient’s hybrid identity management and his/her attempt to regulate the interference of the household in the disease management. The results deepen our understanding of how the degree of HIV/AIDS competence present in a PLWHA’s household affects the manner in which the CHW can perform his or her job and the associated benefits for the patient and his/her household members. In this respect, a household with a high level of HIV/AIDS competence will be more receptive to treatment adherence support, as the patient is more likely to allow interaction between the CHW and the household. In contrast, in a household which exhibits limited characteristics of HIV/AIDS competence, interaction with the treatment adherence supporter may be difficult in the beginning. In such a situation, visits from the CHW threaten the hybrid identity management. If the CHW handles this situation cautiously and the patient–acting as a gate keeper–allows interaction, the CHW may be able to help the household develop towards HIV/AIDS competence. This would have a more added value compared to a household which was more HIV/AIDS competent from the outset. This study indicates that pre-existing dynamics in a patient’s social environment, such as the HIV/AIDS competence of the household, should be taken into account when designing community-based treatment adherence programs in order to provide long-term quality care, treatment and support in the context of human resource shortages.

## 1. Introduction

In order to guide patients successfully along the HIV/AIDS care continuum from testing and linkage to pre-antiretroviral treatment (ART) care, treatment initiation, treatment adherence and retention in care, sufficient attention must be paid to the psychosocial dimensions of chronic disease care [1–3]. However, while efforts to scale up HIV treatment and care have been successful, the care needs of this growing patient group have put pressure on health systems with pre-existing weaknesses, such as the shortage of health workers [4]. In response, community support is being mobilized as a form of task-shifting in order to provide additional care for patients to support them to comply with treatment guidelines in resource-constrained contexts. A broad array of community support initiatives have been identified [5–7] and found to be effective in providing psychosocial support [8–11].

Community-based support moves care closer to the person living with HIV/AIDS (PLWHA) and his/her social environment [8, 11–13]. Because responding to the long-term challenges of HIV/AIDS happens in continuous interaction between the individual and his/her immediate environment [14], the micro-social context surrounding the patient inevitably affects the efficacy of community support initiatives [15]. However, interventions largely ignore the social context in which they are implemented [16, 17]. Consequently, research is required to investigate community-based support within its context [15, 18–21]. In the words of Nhamo, Campbell and Gregson (2010): “there is an urgent need for HIV/AIDS programmers to develop understandings of the way in which the pre-existing social dynamics of their target communities might facilitate or hinder their efforts” [22].

The socio-ecological theory provides a framework for investigating interconnected environments in which disease management occurs [23–26]. Besides specifying different environmental levels of influence, socio-ecological models emphasize the mutual influence and dynamic interplay among these levels [26–30]. Inspired by socio-ecological theory, this article argues that in exploring the potential role of community-based initiatives in individual patient’s ART adherence, the intermediate level of patients’ households should be taken into account [14, 30, 31]. Recently, the pioneering work of Wouters et. al. (2014) showed that peer adherence support interventions have different impacts on patient-level outcomes depending on the family or household context in which the patient lives. Limited research has thus far been conducted on the intermediate role played by households, heightening the need for a full exploration of the mechanisms by which patients’ social environments hamper or facilitate the impact of community-based adherence support programs on patient-level outcomes. This article aims to address this need by responding to the following research aim: *examining the way in which HIV/AIDS competence in the household hampers or facilitates community-based treatment adherence support*.

## 2. HIV/AIDS Competent Household

According to the socio-ecological framework, the intermediate household level should also be the focus of research when linking individual patients to community-based adherence support. We define a household as a “co-residential unit, usually family-based in some way, which takes care of resource management and primary needs of its members” [32]. In our study, the key attributes defining a household are spatial proximity and day-to-day interaction, since these characteristics have been found to be crucial to addressing the day-to-day primary needs of household members, such as care in the context of HIV/AIDS [32, 33].

Based on the same data set used in this article, Masquillier et al. (2015) show that households have the potential to serve as HIV/AIDS competent environments in which patients can be supported on the care continuum in a sustainable manner [34]. HIV/AIDS competency reflects the idea that people’s decision to choose health-enhancing practices, such as HIV testing or safe sexual practices, is influenced by the surrounding social environment and its capacity to actively encourage a lifestyle that fosters good health [35, 36]. HIV/AIDS competent households build a context in which more effective HIV/AIDS management is possible by making prevention and treatment part of daily life in the household. In such a household context, it is easier for the PLWHA to deal with HIV/AIDS-related markers. A feedback loop can also be created in an HIV/AIDS competent household, in which other household members are motivated to be tested voluntarily and counseled and to disclose their status, which is an important step in providing a sustainable answer to HIV/AIDS-related challenges [34].

In the majority of the households surveyed in the Masquillier et. al (2015) study, the PLWHA was the change agent who created awareness and openness about HIV/AIDS. However, what is key to the development of an HIV/AIDS competent household is going further in breaking the silence around HIV/AIDS than simply recognizing the reality of this disease. In a safe social space for dialogue, household members can share the knowledge they have acquired through bridging social capital and talk with other household members in an informed way about the disease and its consequences at the individual and household levels. In this study, bridging social capital is seen as the supportive relationships between the household and outside individuals and organizations [37], with the ability to provide “access to new information and resources, enhancing people’s actual control and improving their ability to solve various problems” [38]. Bridging social capital is required not only to obtain accurate information, but also to access resources from outside that can help households in their efforts to support patients. However, instead of placing the responsibility solely with bridging partners, households must develop their own sense of responsibility about the response to HIV/AIDS and confidence in their own strengths–in a context of solidarity and common purpose–to become truly health-enabling environments for PLWHA.

However, the road to HIV/AIDS competence is precarious and prone to obstacles at various levels. As a result of HIV-related stigma, which can drive HIV/AIDS underground, an atmosphere of secrecy surrounds many households living with the disease [39]. Silence both outside and inside the household can undermine the building of HIV/AIDS competence. While disclosure is a *conditio sine qua non* for starting to build HIV/AIDS competence, it is also a double-edged sword [40]: it opens the door to receiving support, but can also lead to stigmatization, discrimination, and disruption of personal relationships [40, 41]. As a means of managing stigma, PLWHA often adopt a hybrid identity–influenced by the hampering or facilitating context of the household. For example, they may embrace the identity of HIV patients on ART when feeling safe at home, but attempt to present themselves as HIV negative when fear of stigmatization arises [14]. In trying to preserve the hybrid identity, PLWHA may act as *gate keepers* who regulate the interference of their household in HIV/AIDS disease management. The potential consequences of disclosure depend on the pre-existing household context in which the PLWHA lives. Besides positive HIV-related precedents and HIV-related knowledge, positive pre-existing household dynamics such as closeness, an atmosphere of trust, supportive relationships, good internal functioning and open communication–bonding social capital–facilitate disclosure and also provide a good basis for building HIV/AIDS competence in the household. Bonding social capital is considered in this study as strong within-household support, which is important in mobilizing solidarity [37, 42].

HIV/AIDS competence can be thought of as a continuum, sensitive to household dynamics, along which households are positioned and can progress. This progression from less to more competence occurs as households change, opt for health-enhancing behaviors and accumulate the aforementioned characteristics that enable them to better support PLWHA. In what follows, we will explore the interaction between households located at this HIV/AIDS competence continuum and community-based adherence support.

## 3. Methods

We used a combination of interviews, participatory observations and focus group discussions in order to address the following research aim: *examining the way in which HIV/AIDS competence in the household hampers or facilitates community-based treatment adherence support*.

### 3.1 Setting

The fieldwork was conducted in the Mitchell’s Plain/Klipfontein township, an impoverished and densely populated area on the periphery of Cape Town, with a population of 654971, predominantly black and colored. This area is confronted with severe social and economic challenges. Substance abuse, poor schooling and high unemployment are exacerbated by massive population growth [43]. The setting also has high crime rates, especially with regard to murder, rape and drug-related crime [44]. These social and economic challenges translate in health-related challenges. The township is characterized by an HIV prevalence of 19.1%; the number of TB cases is also one of the highest in the country, with 26 658 cases reported in 2011 [45]. This township on the outskirts of Cape Town consists predominantly of informal dwellings and the majority of our respondents had no formal street address.

### 3.2 Data collection

Fieldwork was conducted between February and May 2014 in the township Klipfontein/Mitchell’s plain on the outskirts of Cape Town. During the data collection, the criteria for ART initiation in PLWHA were to start when their CD4 cell count was below 350 cells/μl [46]. At the time of the data collection, TB was also included in South Africa’s National Strategic Plan on HIV, STIs and TB, 2012–2016 [47].

The CHWs we followed during our participatory observations were selected by the NGO. With the aim of minimizing interruption to service provision, the CHWs were contacted for participation in the study in collaboration with district coordinators from the NGO in question. CHWs who were available at the time of the study, who were proficient in English and who agreed to participate took us along on their daily visits to the houses of patients. The patients’ homes formed the setting for the participatory observations performed during treatment adherence support visits from CHWs. Before starting the observation, the CHWs assessed whether the research team was welcome and whether the people present were aware of the patient’s HIV status, in order to avoid inadvertent disclosure and to protect patients’ privacy. If the research team was welcome, the purpose of the study, its design and aspects such as voluntariness and confidentiality were explained in an understandable way to the patients. This information was also distributed by means of an information leaflet–available both in English and the local language. To prevent the inadvertent disclosure of study participants’ HIV status, and to make them as comfortable as possible, participants were permitted to choose the time and place of the interview. Informed written consent was obtained at the first visit of the patient, for the audio recordings and for the publication of the findings. Ethical approval was granted by the Ethics Committee of the University of the Western Cape (13/10/55).

During participatory observations, 13 CHWs were followed on their daily visits to patients in providing community-based treatment adherence support in the townships on the outskirts of Cape Town. Of the 73 houses visited, 48 persons living with HIV/AIDS or their treatment buddies were home for the community-based adherence support session. To minimize the impact on the house visit, the researcher adopted an ‘observer-as-participant’ role. In this respect, the respondents never encountered the research team before the study. In this role, the researcher “is known and recognized, but related to the ‘subjects’ of study solely as a researcher” [48]. Field notes were taken during and after each house visit, including a description of the particular setting, enumeration of the participants, and a description of behaviors and interactions. After every field visit day, the observations were discussed among the principal investigator (CM) and the fieldworkers, who were present at the same observation, to cross-check each other’s findings in order to discover and eliminate inaccuracies–aiming to attain validity [48]. While the principal investigator (CM) was a white, European, middle-class female sociological researcher, the black male and black female fieldworker in this study spoke the respondents’ local language, lived in a neighboring township and shared a common culture. The number of people present during the observations and interviews was determined by the size of the house and the respondent’s preference.

Of the 48 people observed during their community-based adherence support sessions, 41 agreed to participate in an interview on one of the subsequent days. Of these, 9 cancelled the interview, so that 32 people living with HIV/AIDS were interviewed using a semi-structured interview guide adapted according to lessons learned from pilot interviews and observations. Furthermore, data collection and data analysis phases were alternated to inform subsequent interviews and to assess when data saturation had been reached. Interviews were conducted by the principal female investigator (CM), who has experience with these research methods, assisted by one male and one female translator. Interviews were semi-structured to ensure that the same topics were covered in each interview, while also allowing unanticipated material to emerge. First, respondents completed a short interviewer-administered survey to provide basic socio-demographic information, before participating in the qualitative semi-structured interview. The topics explored during the qualitative interview included HIV testing, disclosure, treatment adherence support, among others. The interviews lasted between half an hour and one and a half hours. All interviews but one were audiotaped, allowing us to produce a detailed transcript of the interviews. These transcripts ensured accuracy of what was said and served as the basis for data analysis.

Finally, four focus group discussions were held with 36 of a total of 39 CHWs working for the NGO based at the health facilities included in the study. The principal female investigator (CM) conducted the focus group discussions, assisted by one male and one female translator. Topics that had emerged during the observations and interviews, such as how the CHWs support the patient and how they interact with the social environment of the patient, were discussed in more detail during the focus group discussions.

The findings from the various qualitative research methods we used, allow us to look at the same topic from different angles, rendering the results more valid. Thus, data triangulation is achieved [49]. Besides ‘data triangulation’ and ‘investigator triangulation’, ‘respondent validation’ also enhances the quality of our data. The researcher’s understanding of the data collected in this particular social setting was tested against the perceptions of members of that setting, namely the CHWs, who participated in the focus group discussions. More specifically, the participants of the feedback sessions were invited to give their critical reflections on the findings, which were presented in an understandable way to the participants.

### 3.3 Sample

To be eligible to take part in this cross-sectional study, patients had to be eighteen years or older, HIV positive, and participating in the treatment adherence support program of a particular non-governmental organization (NGO). Under the authority of the Western Cape Provincial Department of Health, the NGO in question trains community health workers (CHWs) to provide adherence support to patients receiving prophylaxis, ART and/or TB treatment. During the treatment adherence support program, the first visit of the CHW to the patient’s home includes a home assessment mapping the social situation of the patient, which is then taken into account when a multi-disciplinary team discusses the patient’s enrolment in the free public ART care. More specifically, during the home assessment the social and housing conditions of the patient are recorded, such as disclosure of HIV and/or TB status to household members, drug and substance use in the household, HIV status of other household members, among others–using a standardized form. Patients receive a monthly supply of ART tablets combined with home visits from CHWs. The aim is to visit the patient weekly during the first month of treatment and subsequently on a monthly basis until viral suppression is achieved.

All but one of the CHWs participating in this study were female. Of the 32 patients interviewed, 10 were male. All patients and CHWs were black and all spoke English and/or a local language. When assessing the highest level of education achieved, the majority of the patients had enjoyed some or completed secondary education. On average, the respondents were 35.6 years old, ranging from 21 to 59. Households had an average of four members. 11 PLWHA in this sample were living with their partner, nine were in a relationship but not living with their partner, and twelve respondents were not in a relationship. Except for two patients, none of the respondents had a paid job. Five PLWHA were receiving a disability grant, while nine were waiting on the response to their application for this grant.

All patients received visits from a CHW to help with adherence. Ten patients had previously defaulted from ART, one of whom was still not taking treatment at the time of the interview. Treatment duration ranged from less than a month to more than 6 years on ART. Most patients were receiving the fixed dose combination. 17 patients reported experiencing side effects from their treatment. 13 of the patients were on both ART and TB treatment.

### 3.4 Data analysis

The recordings of the interviews and focus group discussions were transcribed verbatim and, when necessary, translated into English. A sample of translations was back-translated into the local language for a quality check. Transcripts, moderators and observatory notes were imported into NVivo, version 10. Data collection and data analysis phases were alternated to inform subsequent interviews and focus group discussions and to assess when data saturation had been reached. Coding and analysis were carried out concurrently.

After conceptualizing an HIV/AIDS competent household, a summary of which can be found in section 2 and a detailed description of which can be found in an article by Masquillier et. al. (2015) [34], the analysis focused on the interaction between the community health workers and the patient’s social environment. Data was analyzed carefully by reading and rereading the field notes and transcripts of interviews and focus group discussions. The analysis was performed in accordance with the Grounded Theory procedures described by Strauss and Cobin [50]. First, the data was open coded. In this phase of data analysis, primary information categories which remained close to the original data were constructed. Codes for a sample of transcripts were compared with another researcher’s codes and similarities and differences were discussed. These open codes were then categorized in the axial coding phase to identify any patterns or regularities that emerged, such as the challenges experienced by CHWs when making their visits and the advantages for patients and household members. In the subsequent phase of selective coding, the categories which emerged from axial coding were integrated. For instance, we investigated the relation between the aforementioned axial codes and the various characteristics of an HIV/AIDS competent household. Concepts were refined systematically as more data were collected and analyzed. During this process, special attention was paid to remaining close to the data gathered.

## 4. Results

The analysis indicates, firstly, that community health workers do not work in a vacuum in the community. Secondly, we see that the degree of HIV/AIDS competence present in the household influences both the work of the CHW and the associated benefits for patients and household members.

### 4.1 Community health workers do not work in a vacuum in the community

CHWs often live in the communities they serve. They are active in different locations in their community, they perform a range of different tasks, and they have a number of different roles. Besides offering treatment adherence support in the homes of patients, the CHWs surveyed in this study also participate in education and awareness campaigns in the clinic’s waiting room. Furthermore, they assist in “door-to-door” health promotion visits during which they inform people about health-related topics and invite them to be tested at a mobile testing site as part of outreach programs in the community. Moreover, they also revisit patients who have defaulted on their treatment. Due to these various roles and tasks, their presence in the community is often associated with HIV/AIDS. Receiving a visit from a CHW is often a marker of HIV/AIDS, as these CHWs testify:

*“Even if you know that family and you are only going there for a social visit*, *the neighbors will think that there’s an HIV person in that household since they know what you do at the clinic and community*. *People always have something to say*, *they always talk*, *especially when they know your role*. *Even if you are just having a conversation with someone on your street*, *then they start asking and think that you are talking about the clinic*, *work*.*” (Community health worker*, *health facility 2)*

*“They know that when you saw the CCWs [community care workers] in the community and go to the house*, *the AIDS is smoking there” (Community health worker*, *health facility 3)*

Our results indicate that the association of the CHW with HIV/AIDS makes care more easily accessible for members of the community. For instance, for some patients new to the area, the CHW acted as a guide to accessing the services they needed:

*“Another day*, *the other lady came to my house and said ‘I heard that you are working at the clinic*. *Sometimes when you pass they are gossiping because some have HIV’*. *They were gossiping that day*. *So she decided to go into my house herself*. *She said*, *‘I was taking ARVs in the Eastern Cape*, *so now here I have defaulted for four months*, *I don’t know what I must do now*?*’*.*” (Community health worker*, *health facility 2)*

However, this association also aroused stigmatizing feelings and threatened the hybrid identity management of some PLWHA in the surrounding community, as it may stimulate or force the disclosure of their status. To limit these unintended negative consequences, the CHWs develop various strategies to conceal their role as a CHW outside the health facility. While the CHWs wear the uniform of the NGO in the clinic and the community outreach programs, they are not identifiable as health care workers of this NGO while walking to patients’ homes or visiting patients. Patients who are in need of help have few issues with the uniform, but others prefer the CHWs to visit in regular clothes:

*“I don’t have any problem with her visiting me when she is in her clothes*, *not wearing the uniform*. *I do have problem when she is in uniform*. *[…] It is because people talk*. *They become suspicious that she is doing home visits for people who are living with HIV” (Female PLWHA*, *38)*

### 4.2 Interaction between the patient’s household and the community health worker

As the CHWs bring care closer to patients’ homes, there is a higher chance of interaction between CHWs and patients’ household members than is the case with regular health care provision at the facility. Results suggest that, even without the uniform, the presence of the CHW is a marker of the disease. In this regard, the visits can complicate patients’ hybrid identity management. To preserve their hybrid identity, and depending on the HIV/AIDS competence in the household, patients may try to act as gate keepers by involving the household in the treatment adherence support sessions only when they feel safe as PLWHA on ART in the home.

Identifying whether the CHWs’ treatment adherence support visits are welcome is challenging, especially the first time. As the results indicate, CHWs often need to ask neighbors for help finding the address, do not know what the patients look like and are unaware of the household situation. In this regard, the home assessment performed during the first visit is a useful tool for mapping these issues for future visits so that the CHW can employ strategies to hide the purpose of the visits if needed.

*“I enter into the veranda and opened the house door*, *that guy knows me and I didn’t know him*, *I was about to ask for the person and he just chased me away*. *It was like there’s a bomb that will explode*. *I asked ‘why*?*’*, *he said ‘I know you are from Mzamomhle’*, *and he said ‘don’t enter into my house because my girlfriend is there and I don’t want her to know anything about my health’ […] Up until sister [nurse] said ‘no*, *leave him*, *he’s a long time defaulter’*. *Now he has MDR [multi-drug resistant TB]*, *so I’m no longer going there*.*”(Community health worker*, *health facility 2)*

#### 4.2.1 Living in a household with a low level of HIV/AIDS competence

When patients do not disclose to all household members, a burden of secrecy is created. Results suggest that for some patients, the only way to preserve their hybrid identity is to attempt to avoid the treatment adherence support visits, as they are often a marker of HIV/AIDS. For instance, patients may refuse permission for the visits during the counseling sessions; provide incorrect contact information; pretend to be someone else when the CHW first visits; or ask to change the location of the visits. Other patients in the study received their CHW at the house of a relative or friend to avoid having the visits at home or because they had been tested in a clinic outside of their own community in order to avoid being recognized as living with HIV/AIDS in their community. Often, visits are not completed successfully in such situations.

*“I’ve got a client who also did not disclose to her daughter*. *She said to me*, *‘If you come here and my daughter is here*, *you better not do anything and go back*. *You better talk as if you know me from the church*, *because I know my daughter*, *she drinks alcohol*, *so when she’s drunk she’s gonna tell everyone and she’s going to stand outside and shout at me and tell everyone that I’m HIV positive*. *So that’s the reason why I don’t tell her’*. *I remember one time I went to that house and the daughter was there*, *she then winked and whispered to me ‘Don’t say anything*. *You must come another day’*. *So this stigma thing is there*, *because they are ill-treated by their own families*.*” (Community health worker*, *health facility 4)*

When patients do not want the CHWs to perform the visit, they try to communicate this by winking, whispering or making excuses about why this is not the right time for the visit, and asking the CHW to come back another day. Patients who accept the treatment adherence support visit might need to disguise it if there are members of the household present who are unaware of the HIV status of the PLWHA. Our results demonstrate that patients employ strategies to conceal the reason behind the visits, such as disguising their ART when giving it to the CHW; giving only the TB treatment to the CHW; making up a story about the CHW; meeting in a private room, such as the bedroom; or by going to the CHW’s own house.

Besides patients, CHWs themselves also employ strategies to conceal the actual purpose of their house visits. The CHWs surveyed in our study may stop the treatment adherence support session if someone enters the house; have the session in a private room like the bedroom; ask for privacy when other people are present in the house; make up a story, such as pretending to be selling various commodities; and use TB as a disguise. However, these strategies are not always effective, since some members of the community nevertheless associate the CHWs with the clinic. In responding to such a situation, some CHWs identify themselves as health educators who come to inform the entire family about a health-related topic, such as TB, which most consider to be less stigmatizing.

*“I had to pretend now to be someone who came looking for gloves*, *and now I did end up not doing anything because there were kids in the house*. *Even the second day when I went*, *there were kids in the house and she didn’t want anything that was going to come out*, *like medicine or whatever is related to the clinic*. *So I ended up doing nothing*, *I left*.*” (Community health worker*, *health facility 4)*

#### 4.2.2 Living in a household with a high level of HIV/AIDS competence

When living in a more HIV/AIDS competent household, it is easier for PLWHA to cope with HIV/AIDS-related markers, such as ART or CHWs’ treatment adherence support visits, rather than deploying strategies to keep these a secret. In such a situation, the patient–as a gate keeper–will be able to accept the support of the CHW, this bridging social capital, more easily. Sometimes it is even the household members who convince the patient to accept the CHW’s visits. Furthermore, a health-enabling household environment facilitates the work of the CHW. For example, the CHW no longer needs to find excuses; a household member can hand over the medication for a pill count so that the CHW does not have to return if the patient is not home during the first visit; and a household member can direct the CHW to where the patient is if he or she is not home.

*“When she comes we try to make sure that there is someone at the house if I am not there*. *[…] If I am not there*, *someone is around here to help her*. *Because they know where the tablets sits*. *[…] It’s better that she comes here*, *at least we have an open relationship with everyone in the house*.*” (Male PLWHA*, *24)*

### 4.3 Benefits of treatment adherence support for patients and their household members

While CHW visits help at patient level, they also have the potential to create involvement and provide support at household level. Listening to the history of the patient and his or her household and witnessing the day-to-day household dynamics, the CHW can implicitly diagnose where the household is located on the HIV/AIDS competence continuum. When the patient–as a gate keeper–allows interaction between the CHW and the household, the CHW–as bridging social capital–might be able to help the household increase its HIV/AIDS competence.

#### 4.3.1 Living in a household with a low level of HIV/AIDS competence

While it is often more difficult to help patients living in less HIV/AIDS competent households, there is a bigger potential for help. As these patients receive less support from their households, the CHWs can play an important role in supporting the patients along the care continuum. CHWs help patients to overcome various barriers that make adherence more difficult. Being bridging social capital, the CHW can help the patient access resources from outside, such as condoms. Furthermore, the CHW sometimes acts as a bridge between the patient and official institutions. For instance, CHWs may inform patients about the status of files drawn up by social workers. Moreover, CHWs help patients with housekeeping if the patient is too weak and provide both material support (e.g. financial resources or food) and emotional support.

*“I will be very hurt [if the visits from the CHW stop]*, *because some of the things I want to say I can’t say them to my father*, *and sometimes I don’t have airtime to call my mother” (Female PLWHA*, *22)*

Furthermore, the CHWs in this study also helped to involve households in disease management, for example by encouraging patients to bring a treatment buddy to the counseling sessions, which is required to start ART. When CHWs conclude that the house the patient is living in is not a supportive environment, they try to encourage the patient to move to another house. Similarly, in households where there is no awareness of the disease among household members yet, the CHWs try to encourage patients to disclose their status.

*“I tried to convince him but he says ‘no*, *I know my wife*, *what kind of person she is*. *So if I can tell her that*, *maybe she will leave’*. *They are worried of being rejected by the wife or husband*. *And the wife also said the same story up until I arranged*, *I said ‘I can be here when you disclose to your wife’*. *And also I said to the wife ‘I won’t mind’*. *I arranged one day for them*.*” (Community health worker*, *health facility 2)*

The CHWs surveyed in this study not only help with recognition of HIV/AIDS in the household, but also guide the household towards increasing HIV/AIDS competence. This is on the condition that patients, as gate keepers, allow their interaction. Depending on the pre-existing household dynamics, such as a culture of open communication and a climate of trust, the presence of the CHW has the potential to help the household in the phase of exchanging and sharing knowledge and prevention skills. In such a climate of open communication, the PLWHA might communicate the knowledge they have gained about HIV/AIDS and prevention skills from the CHW to other household members, or other household members may ask questions of the CHW regarding their own health or that of the patient.

*“Other families do not know everything*. *They usually ask from us*, *if this is right or not*, *although they are supporting the patient” (Community health worker*, *health facility 1)*

However, in order to become more HIV/AIDS competent it is important that patients and their households feel responsible and confident that they have the strength to effectively respond to the challenges of living with HIV/AIDS, instead of regarding it passively as the responsibility of the bridging partners. One CHW mentioned that the support sometimes made other household members feel less involved, which had a negative effect on the sense of ownership and responsibility in the household. Furthermore, while some patients indicated that they would visit the CHW at home to continue support if the visits stopped, others thought they might stop or change the way they took their medication, as this patient testifies:

*“She encourages me to take my treatment*. *If she stopped visiting me*, *I would be sad because maybe I would be lazy to take my pills*, *telling myself that nobody will ever notice that I don’t take them regularly” (Female PLWHA*, *21)*

The CHW tries not only to improve HIV-related behavior, such as treatment adherence and HIV-preventive behavior, but also to help patients incorporate a healthy lifestyle in their daily lives, for example, by advising them against mixing alcohol and ARVs:

*“There is time that I run away from her [CHW] when I’m drunk*. *Because I don’t take medication when I’m drunk and I know she is going to shout at me not to take my medication [emotional]” (Female PLWHA*, *30)*

#### 4.3.2 Living in a household with a high level of HIV/AIDS competence

While it is easier for CHWs to offer treatment adherence support when patients feel responsible for their own health and live in HIV/AIDS competent households, the room for improvement is more limited.

*“The family takes the CCW’s [community care worker’s] role*. *Because I think if the family can remind the patient to take his/her medication*, *then everything will be fine” (Community health worker*, *health facility 4)*

Nevertheless, even a more HIV/AIDS competent household needs bridging social capital to access accurate information and resources from outside that can assist the household in its efforts to support the patient. Examples from the study include a CHW who checked whether patients were receiving the correct medication from the clinic and a CHW who formed a bridge between the patient and an official institution. Moreover, household members can also benefit from the CHW’s visits. Examples include a patient passing condoms received from the CHW to her/his household members; a CHW counting medication for a child in the house as well; and a CHW making a referral for household members to go to the clinic for a HIV test or other services needed:

*“I also speak to them [brothers]*: *if they tested*, *how is their life*, *and [whether] they are sure about their status*. *Then they said ‘no sisi*, *we are not sure about our status*, *but we are just encouraging our brother*. *But you can do’*. *And I say ‘can I do referrals so that you can go to the clinic*?*’*. *The two of them said ‘you can do’*, *the third one said ‘no I’m always with him in the clinic so I can just go like that’*.*” (Community health worker*, *health facility 3)*

As members of HIV/AIDS competent households are more confident about making their stories and life experiences public in their communities, even neighbors sometimes benefit from CHW visits:

*“Sometimes she would find me with people and she can explain things to me*, *but she first asks if she can explain things in front of other people*, *and I would say ‘Yes they do know about this*, *please do come and explain to us*, *yes’*.*” (Female PLWHA*, *43)*

## 5. Discussion

Masquillier et al. (2015) show that the HIV care continuum–which determines the pathway a patient will take after a positive HIV test [51, 52]–exists almost entirely independently of the household [34]. However, home visits from a CHW providing treatment adherence support move the HIV care continuum closer to the PLWHA and his/her social environment. The aim of this article was to assess how dynamics in the patient’s household interacts with the community-based treatment adherence support.

Despite the fact that CHWs try to present themselves neutrally when making home visits, our results indicate that the presence of a CHW is often seen as a marker of HIV/AIDS. In line with Goffman, markers of the disease transform HIV from a hidden to a visible disease, allowing people to label themselves or others as HIV-positive. This can trigger stigmatizing feelings or behavior [41, 53–55]. In this regard, despite the advantages of bringing care closer to the community, community-based treatment adherence support also poses challenges for both patients and CHWs–depending on the HIV/AIDS competence of the household, among other factors. In response, both patients and CHWs employ strategies to disguise the real intention of the visits of the CHW. From a patient’s perspective, this can complicate hybrid identity management. It can also make it more difficult for the patient to act as a *gate keeper*, i.e. to regulate the household’s role in disease management. As theorized by Goffman, individuals possessing stigmatizing attributes attempt to mitigate stigma by manipulating the information they share with others [55]. This study corroborates the findings of Remien et al. (2013), Ciambrone et al. (2006) and Gusdal et. al. (2011), who found that confidentiality concerns were a potential barrier to participation in community-based adherence support programs [56–58]. In the words of Gausset *et*. *al*. (2012): “anonymity and confidentiality do at times shield PLHIV [people living with HIV] from stigmatization, but since it cannot be guaranteed by the current system, people (especially men), attempt to remain anonymous by avoiding CBO [community-based organizations] or seeking treatment in places where they are not known, which can delay their search for treatment” [59]. From the CHW’s perspective the association with HIV/AIDS influences how CHWs are able to do their jobs, similarly to the community-based HIV/AIDS care providers surveyed in the study of Wools-Kaloustian et. al. (2009), who found that community care providers defined themselves as health counsellors in order to avoid the AIDS label and associated stigma [60].

This article shows that the household environment of the patient interacts with the community-based treatment adherence support in different ways. On the one hand, a household located at the supportive end of the HIV/AIDS competence continuum will be more receptive to treatment adherence support, as the patient is more likely to allow interaction between the CHW and the household. In such a household context, PLWHA will have less fear of stigmatization and be more likely to embrace the identity of an HIV patient on ART [34]. This household context will facilitate the CHW’s work. However, as the household itself acts as a supportive and health-enabling environment for PLWHA, this support might have limited added value for the patient. Nevertheless, our results indicate that household members also benefit directly or indirectly from this bridging social capital. For instance, a household which is a social space for dialogue and critical thinking can facilitate the dissemination of information from the CHW among household members. On the other hand, in a household which exhibits limited characteristics of HIV/AIDS competence, interaction with the treatment adherence supporter may be difficult in the beginning. Out of fear of stigmatization, prompted by negative pre-existing household dynamics or negative HIV-related precedents, PLWHA will try to present themselves as HIV negative by adopting a hybrid identity. If a patient decides not to disclose to some or all household members, a burden of secrecy is created [34]. In such a situation, visits from the CHW threaten this hybrid identity management. If the CHW handles this situation cautiously and the patient–acting as a gate keeper–allows interaction, the CHW may be able to help the household develop towards HIV/AIDS competence. This would have a more added value compared to a household which was more HIV/AIDS competent from the outset. For instance, the help offered to patients by the CHWs surveyed in this study during the phase of recognizing the reality of HIV/AIDS in the household echoes a key finding of the study of Root and Whiteside (2013), who found that a “care supporter’s agility at creating safe disclosure settings was felt to be one of their most important practices” [61]. However, our results also demonstrate that some household members become less involved when patients are receiving the CHWs’ support. Similarly, Nhamo et. al. (2010) reported that interventions by outside professionals sometimes undermine local ownership unintentionally [22].

This study is subject to several limitations. First, we acknowledge that the selection bias present in this study means that the findings cannot be generalized to the most vulnerable patient groups. Patients who avoided treatment adherence support by not agreeing to the visits during counseling or by providing the incorrect address could not be reached during the participatory observations and interviews. Similarly, to avoid inadvertently disclosing a patient’s status, and to protect patients’ privacy, the CHWs did not perform treatment adherence support visits when it was unclear whether the people present were aware of the patient’s status. Furthermore, during the time patients were given to reflect on their decision to participate, nine patients cancelled the interview. Three of these patients were no longer willing to participate in the interview, four were not at home on the date of the interview and we were unable to track them, and two were unable to attend because of work. These patients might also have been typical of the group that is most difficult to reach in the treatment adherence support program. Second, no household members were asked to participate in the study, with the aim of safeguarding the confidentiality of the PLWHAs. However, some household members did participate in the interview spontaneously, which may be an indication of the openness to HIV/AIDS in that particular household.

This article extends the notion of HIV/AIDS competence to the household. As one of the first studies to incorporate the intermediate role of the HIV/AIDS competent household, this article aimed to investigate comprehensively the impact of community-based adherence support on a patient’s care continuum. Future research could also advance our understanding of the importance of a patient’s social environment by applying the lessons learned from HIV/AIDS-related interventions to other chronic and non-communicable diseases. In this respect, further research could make interesting progress by investigating the potential for making HIV/AIDS competent households into more comprehensive health-competent households. Moreover, our study provides additional support for Root and Whiteside’s (2013) recommendation that research should be carried out to determine whether community home-based care services create ripple effects between clients and household members [61]. Our findings corroborate Bogart and colleagues’ statement that “interventions to improve adherence to HIV treatment may also need to take into account the household context of felt stigma and disclosure in order to be effective” [39]. In this regard, the current study shows that, in order for an ART program to be effective and sustainable, we must capitalize on the intermediate level of the household unit to optimize the impact of community-level adherence support. Further qualitative in-depth research is imperative to improve our understanding of the (economic) role and social positioning of the PLWHA within the HIV/AIDS competent household. Furthermore, analyzing HIV/AIDS competent households and the interaction with the CHW through a cultural and gender lens provides an interesting path for further research. Future research could make interesting progress by analyzing the studied interaction in the long run, aiming to capture the dynamic aspects of households more in depth. Furthermore, in accordance with socio-ecological theory, this study highlights the fact that interventions do not happen in a vacuum. In this regard, this article responds to the research need formulated by Kok et. al. (2015) in their systematic review, where they state that “research which includes adequate descriptions and analysis of context is needed to provide evidence on the influence of contextual factors on CHW performance as the current evidence is not sufficient to assist policy makers to develop CHW programs and interventions that anticipate or make use of context” [62]. More specifically, this article adds to the limited evidence available on the influence that disease-related stigma has on the performance of CHWs.

From a practice and policy perspective, this study provides us with multiple insights. First, the results show that in the phase of developing interventions, policy makers and researchers should look at how treatment adherence support can be made more sensitive to the context in which it is implemented. This insight is in accordance with Pawson and Tilley’s argument that the context in which a program is realized is as important for its success as are the technical details of this program [22]. The results indicate that there is a need to focus more on the household environment in HIV/AIDS care–in line with the findings of Garner et al. (2007) and Grey et al. (2006) [63, 64]. In the words of McNairy and El-Sadr (2012), support measures should “be tailored to the social context and characteristics of the specific patient populations they serve” [51]. Second, the results demonstrate that generic HIV/AIDS care does not necessarily facilitate treatment adherence among the most vulnerable patient groups, particularly those lacking a supportive HIV/AIDS competent household context. Our study adds further weight to the recommendation of Weihs et. al. (2002) that “customization of interventions on the basis of family needs and styles might optimize outcomes” [23]. This study also supports the call for using differentiated approaches for patients at low or high risk of being lost along the care continuum. This is essential, as antiretroviral treatment cohorts continue to expand, as does the need to retain patients over time with limited human resources. Those who are supported by their social environments and feel responsible for and confident about their ART might not experience as much benefit from the treatment adherence support provided by the CHW. Following the first few visits from a CHW, strategies are needed to guide these patients towards other forms of support that draw on the patient’s sense of responsibility, such as peer support groups [65] or community ART groups [66]. Such a differentiated approach could have a positive effect on the resources made available to patients who are identified as being in need of support during the CHW’s first visits. These patients, often living in less HIV/AIDS competent households, could benefit greatly from support but are more likely to fall through the cracks. As articulated by Spire et al. (2002) and Muñoz et. al. (2011), social support needs are dynamic and unique to each individual, depending on existing networks, among other factors, meaning that “adherence strategies should vary over time to reflect the needs of the individual” [67, 68]. Third, attention should be paid to the fact that CHW support ought to build on a patient’s internal motivation, so that it stimulates his/her sense of ownership and responsibility as well as that of the household. The CHW should become a sort of coach, helping to incorporate disease management in patients’ daily lives so that patients are prepared to adhere to treatment even without the CHW’s support. Fourth, given the high TB/HIV co-infection rates, integrated TB and HIV treatment adherence support is vital. This is reflected in the current South African guidelines, the National Strategic Plan on HIV, STIs and TB 2012–2016. Treatment support for HIV and TB will even be extended to include other health conditions within the current re-engineering of primary health care (PHC). As part of this South African health care reform, it is envisaged that CHWs will join local ward-based PHC teams led by professional nurses [45]. Re-engineering PHC is important, because it will increase coordination and communication between services via CHWs [67]. Additionally, the extension of the CHW’s role to include non-HIV services may also reduce their being perceived as markers of HIV. Future research could make interesting progress by investigating CHW visits that cover a broad range of health care tasks, paying attention to the context in which it is implemented.

## 6. Conclusion

In order to provide long-term quality care, treatment and support in a context of human resource shortages, pre-existing social dynamics in the social environment of the patient, such as the level of HIV/AIDS competence in the household, should be taken into account when designing community-based treatment adherence programs. 
